# Supplementary material for: Taxonomy and Phylogeny of Cystolepiota (Agaricaceae, Agaricales): New Species, New Combinations and Notes on the C. seminuda Complex
Source: J Fungi (Basel). 2023 Apr 30;9(5):537. doi: 10.3390/jof9050537 (PMC10218902; doi:10.3390/jof9050537)
Supplement: Supplementary file 1 [file jof-09-00537-s001.zip › Figure S3.pdf]

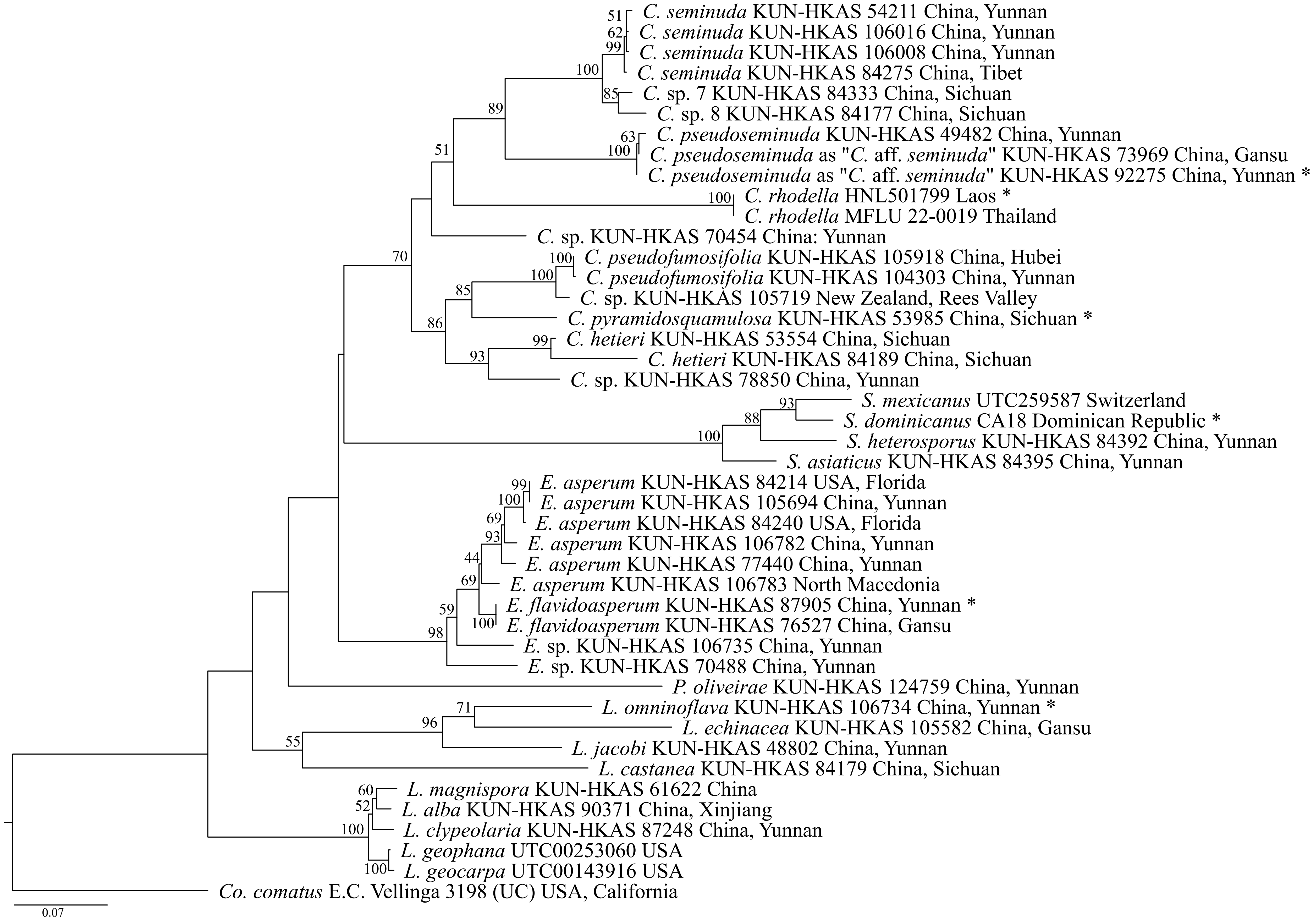

Figure S3. Maximum likelihood tree inferred from *rpb2* sequences of specimens of *Cystolepiota* and related genera. Bootstrap values of ML ≥ 50% are indicated above the branches. Type collections are marked with an asterisk. *C.* = *Cystolepiota*; *Co.* = *Coprinus*; *E.* = *Echinoderma*; *L.* = *Lepiota*; *P.* = *Pulverolepiota*; *S.* = *Smithiomyces*. *Co. comatus* is used as outgroup.
